# Alpha-lipoic acid attenuates heat stress-induced apoptosis via upregulating the heat shock response in porcine parthenotes

**DOI:** 10.1038/s41598-023-35587-6

**Published:** 2023-05-24

**Authors:** Song-Hee Lee, Ming-Hong Sun, Wen-Jie Jiang, Xiao-Han Li, Geun Heo, Dongjie Zhou, Zhi Chen, Xiang-Shun Cui

**Affiliations:** 1 Department of Animal Science, Chungbuk National University, Cheongju, Republic of Korea; 2 College of Animal Science and Technology, Yangzhou University, Yangzhou, 225009 People’s Republic of China

**Keywords:** Embryology, Gene expression analysis, Imaging, Proteomic analysis, Cell death, Environmental impact

## Abstract

Heat stress (HS) is a long-standing hurdle that animals face in the living environment. Alpha-lipoic acid (ALA) is a strong antioxidant synthesized by plants and animals. The present study evaluated the mechanism of ALA action in HS-induced early porcine parthenotes development. Parthenogenetically activated porcine oocytes were divided into three groups: control, high temperature (HT) (42 °C for 10 h), and HT + ALA (with 10 µM ALA). The results show that HT treatment significantly reduced the blastocyst formation rate compared to the control. The addition of ALA partially restored the development and improved the quality of blastocysts. Moreover, supplementation with ALA not only induced lower levels of reactive oxygen species and higher glutathione levels but also markedly reduced the expression of glucose regulatory protein 78. The protein levels of heat shock factor 1 and heat shock protein 40 were higher in the HT + ALA group, which suggests activation of the heat shock response. The addition of ALA reduced the expression of caspase 3 and increased the expression of B-cell lymphoma-extra-large protein. Collectively, this study revealed that ALA supplementation ameliorated HS-induced apoptosis by suppressing oxidative and endoplasmic reticulum stresses via activating the heat shock response, which improved the quality of HS-exposed porcine parthenotes.

## Introduction

Heat stress (HS) is an environmental determinant that adversely affects the productivity of animals^1^, and greatly impairs reproductive performance by decreasing fertility during the summer season^2^. Elevation of ambient temperature adversely affects diverse aspects of pregnancy, including oocyte quality, fertilization efficiency, and successful embryonic development^2,3^. In recent years, the negative impacts of thermal stress in pigs have gradually increased, indicating that genetic selection for reproductive and lean tissue growth results in sensitivity to heat^4^. Although compromised embryo competence induced by HS has previously been reported^3,5^, further studies are required to fully understand the mechanisms in heat-stressed porcine embryos and to assess their therapeutic potential.

Mammalian cells respond differently to environmental stressors, including oxidative stress response, unfolded protein response (UPR), and heat shock response (HSR), to allow cell survival under unfavorable conditions. In response to HS, reactive oxygen species (ROS) production is increased and oxidative stress is induced, which can lead to apoptosis^5^. These environmental and cellular stresses trigger the accumulation of unfolded proteins in the endoplasmic reticulum (ER), which is termed ER stress. They also activate the UPR as a protective pathway to maintain protein homeostasis^6^. HSR is a fundamental protective mechanism against various stressors, and it normally upregulates heat shock factors (HSFs), which is followed by increased expression of heat shock proteins (HSPs) to suppress protein damage or aggregation^7,8^. The distinction between HSR and UPR allows independent responses to operate during protein restoration with their particular chaperones^6^.

In addition, HS compromised in vitro embryo production by increasing ROS in cows^9^, and induced oxidative and ER stresses that affect the activity of antioxidant enzymes and expression of ER stress-related genes in chicken testes^10^. Moreover, embryos produced in vitro had increased HSP70 mRNA expression after exposure to HS in mares^11^, and pig ovaries exposed to thermal stress showed upregulation of *HSP70*, *HSP40*, *HSPH1*, *HSPA4*, *HSF1*, and *HSF2* genes^12^.

Alpha-lipoic acid (ALA), which is primarily involved in mitochondrial dehydrogenase reactions, has recently gained substantial attention as a potent antioxidant^13^. The therapeutic potential of ALA has been extensively studied in diverse oxidative stress-related diseases^14^. ALA scavenges ROS and provides reduced glutathione (GSH), inhibiting the formation of free radicals to maintain redox homeostasis in cells^15,16^. Moreover, ALA protects the testes against heat damage by suppressing oxidative and ER stresses in chickens^10^, improves embryo quality, and enhances cryotolerance by reducing ROS production in cows^17,18^. Despite numerous studies on the antioxidant effects of ALA, its impacts on heat-stressed porcine parthenotes remain to be elucidated. Thus, the purpose of this study was to examine the protective effects of ALA on HS-induced porcine parthenotes and further assess whether cellular stress and the HSR are associated with this protective process.

## Results

### HS disrupted parthenotes development in pigs

**Figure 1:**
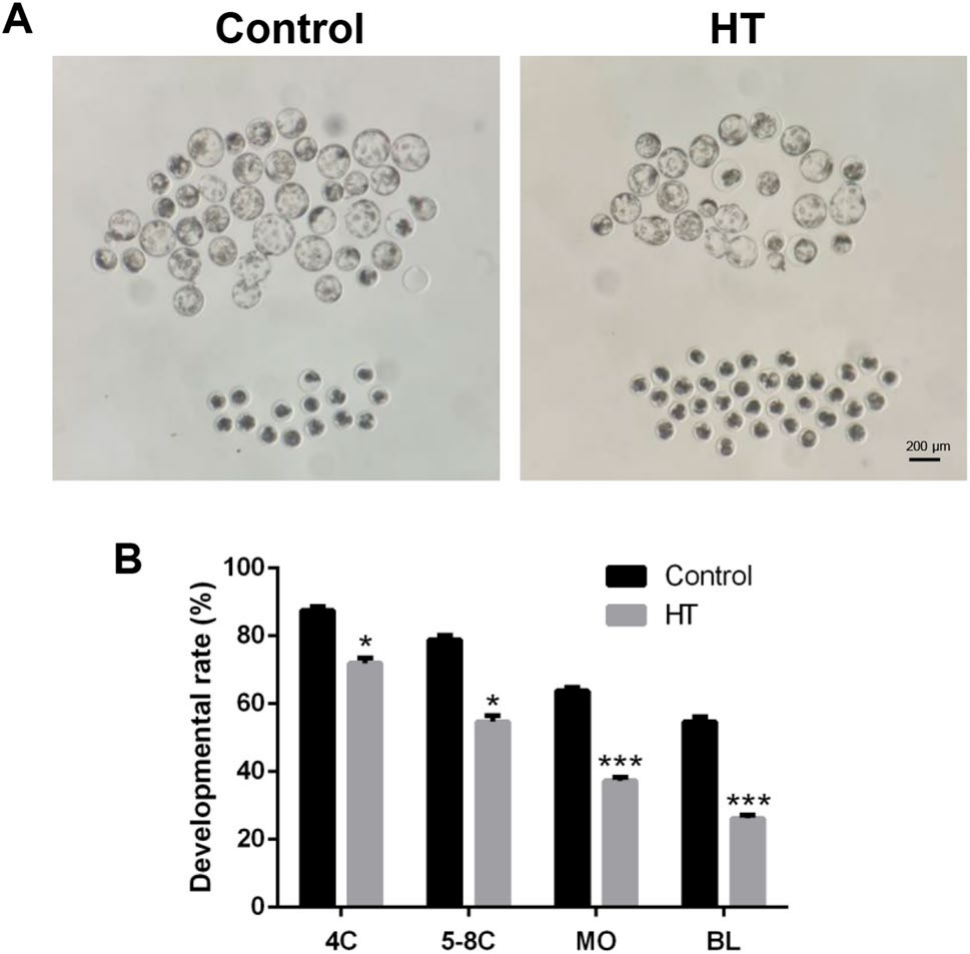
Effect of high temperature (HT) on parthenotes development in pigs. (**A**) Representative images of the parthenotes development in the control group and groups exposed to HT for 10 h. (**B**) The developmental rate from four-cell to blastocyst stage in the control and HT group. *p < 0.05; ***p < 0.001.

To confirm the harmful effects of high temperature (HT) on porcine parthenotes development, we first investigated the development of parthenotes exposed to HT at 42 °C for 10 h. After exposure to HT, the developmental rate from four-cell to morular stage was gradually decreased compared to that in the control group (four-cell stage: control: 87.56 ± 1.09% vs. HT: 71.99 ± 1.53%, *p* < 0.05; five to eight-cell stage: control: 78.85 ± 1.29% vs. HT: 54.73 ± 1.83%, *p* < 0.05; morular stage: control: 63.76 ± 1.07% vs. HT: 37.31 ± 1.1%, *p* < 0.001). Finally, the rate of blastocyst formation was significantly decreased (29.27 ± 2.99%, *p* < 0.01) compared to that in the control group (58.23 ± 4.61%, Fig. 1A,B), indicating that HT disrupts cleavage and consecutive progress during parthenotes development. These results show that HS reduced the potential for the development of porcine parthenotes.

### ALA rescued HS-induced impairment of porcine parthenotes development

**Figure 2:**
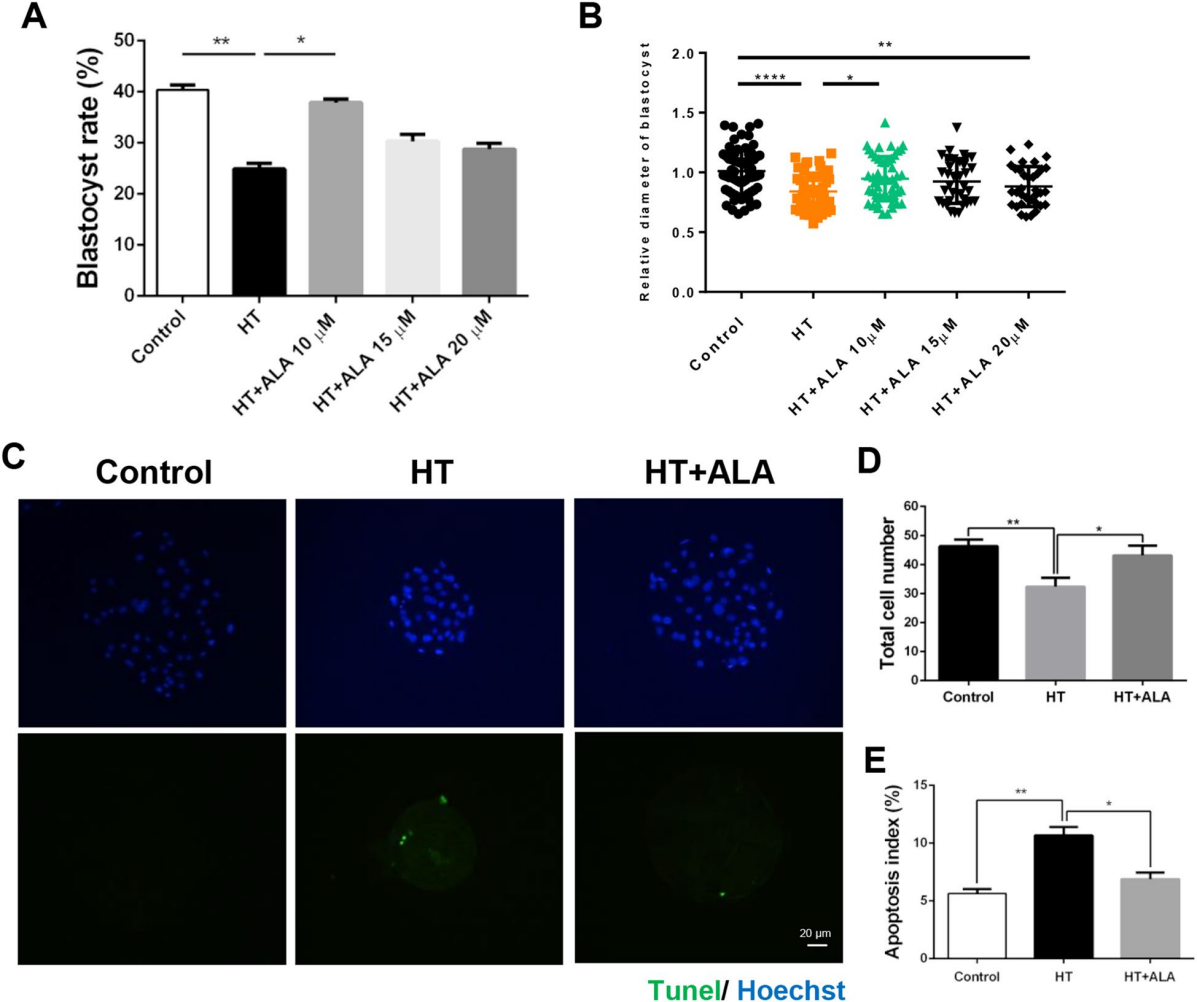
Effect of alpha lipoic acid (ALA) on heat stressed-porcine parthenotes development. (**A**) Rate of blastocyst formation after HT exposure with the addition of 10, 15, and 20 µM ALA. (**B**) Relative diameter of blastocysts after HT exposure with ALA addition. (**C**) Representative images of the total cells and TUNEL-positive cells after HT exposure with ALA addition in porcine embryos. Blue, DNA; green, TUNEL-positive signal. (**D**) Total cell number in porcine blastocysts after HT exposure with ALA addition. (**E**) Apoptosis index calculated by number of TUNEL-positive cells by total cells after HT exposure with ALA addition. *p < 0.05; **p < 0.01; ****p < 0.0001.

To explore the protective effects of ALA on HT-induced parthenotes, we first assessed the rate of parthenotes development following the addition of ALA in a dose-dependent manner. The results showed that the addition of 10 µM ALA partially restored the rate of blastocyst formation compared to the HT group (control: 40.38 ± 0.99% vs. HT: 25.35 ± 1.13%, *p* < 0.01; HT: 25.35 ± 1.13% vs. HT + 10 µM ALA: 35.54 ± 0.95%, *p* < 0.05; HT + 15 µM ALA: 30.33 ± 1.32%; HT + 20 µM ALA: 28.76 ± 1.15%, Fig. 2A). In addition, we evaluated the quality of blastocysts using two methods: assessment of blastocyst diameter and TUNEL assays, which detect the number of dead nuclei. As shown in Fig. 2B, although HT exposure markedly reduced the diameter of blastocysts, treatment with 10 µM ALA partially restored its full diameter. However, addition of 20 µM ALA was diminished the quality in blastocysts (control: 1.00 ± 0.05 vs. HT: 0.83 ± 0.05, *p* < 0.001; HT: 0.83 ± 0.05 vs. HT + 10 µM ALA: 0.94 ± 0.06, *p* < 0.05). Therefore, 10 µM of ALA was used in subsequent studies. Moreover, as shown in Fig. 2C, low blastocyst quality was observed in the HT group, indicating an increase in TUNEL-positive cells and a decrease in the total number of cells. In contrast, ALA addition reduced the number of TUNEL-positive cells and increased the total number of cells, suggesting that it improves the quality of blastocysts under HT (total number of cells: control: 46.38 ± 0.68 vs. HT: 32.43 ± 0.89, *p* < 0.01; HT: 32.43 ± 0.89 vs. HT + ALA: 43.22 ± 0.87, *p* < 0.05; Apoptosis index: control: 5.65 ± 0.39% vs. HT: 10.66 ± 0.73%, *p* < 0.01; HT: 10.66 ± 0.73% vs. HT + ALA: 6.89 ± 0.57%, *p* < 0.05, Fig. 2D,E). Collectively, these results indicate that ALA partially restored the rate of parthenotes development and quality in parthenotes subjected to HT.

### ALA rescued HS-induced oxidative stress in porcine parthenotes

**Figure 3:**
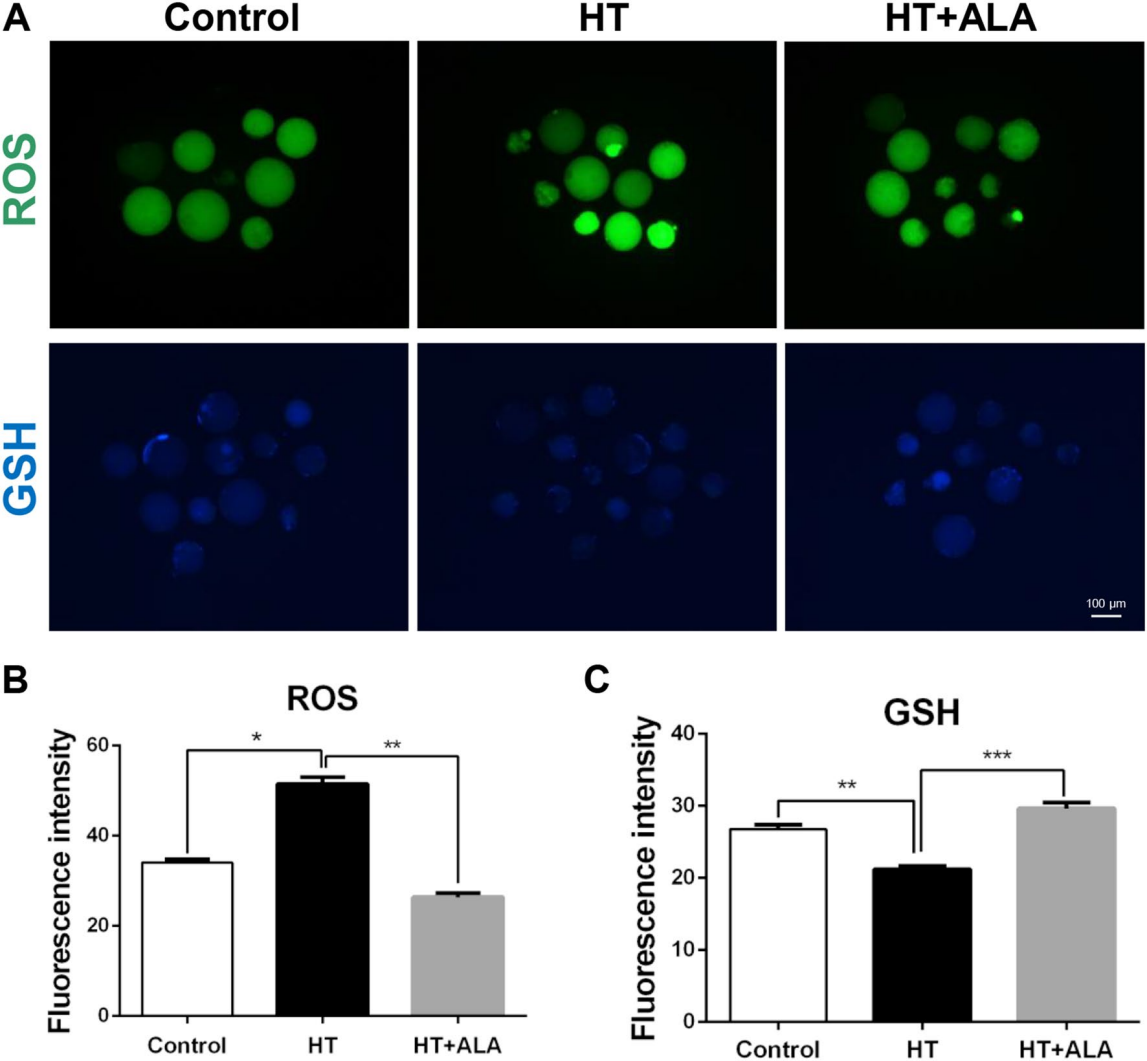
Effect of ALA on oxidative stress in heat stressed-porcine parthenotes. (**A**) Representative images of reactive oxygen species (ROS) and glutathione (GSH) level after exposure in the presence of ALA in porcine parthenotes. Green, ROS; Blue, GSH. (**B**) Relative fluorescence intensity of ROS after HT exposure with ALA addition. (**C**) Relative fluorescence intensity of GSH after HT exposure with ALA addition. *p < 0.05; **p < 0.01; ***p < 0.001.

To evaluate the antioxidant effect of ALA on oxidative stress induced by HT, we first estimated ROS levels after ALA addition. As shown in Fig. 3A,B, HT exposure significantly increased ROS levels, resulting in increased oxidative stress. However, treatment with ALA partially reduced ROS levels compared to the HT group (control: 34.06 ± 0.73 vs. HT: 51.51 ± 1.48, *p* < 0.05; HT: 51.51 ± 1.48 vs. HT + ALA: 26.36 ± 0.93, *p* < 0.01). No difference was observed between the control and HT + ALA groups. We also investigated GSH levels as an indicator of antioxidant capacity under HS conditions. Although exposure to HT significantly reduced GSH levels compared to the control group, ALA addition markedly restored GSH levels compared to the HT group, showing it has a strong antioxidant defense capacity (control: 26.80 ± 0.57 vs. HT: 21.23 ± 0.43, *p* < 0.01; HT: 21.23 ± 0.43 vs. HT + ALA: 29.65 ± 0.79, *p* < 0.001, Fig. 3A,C). These results suggest that ALA addition partially ameliorated the oxidative stress induced by HT in porcine parthenotes.

### ALA rescued HS-induced ER stress in porcine parthenotes

**Figure 4:**
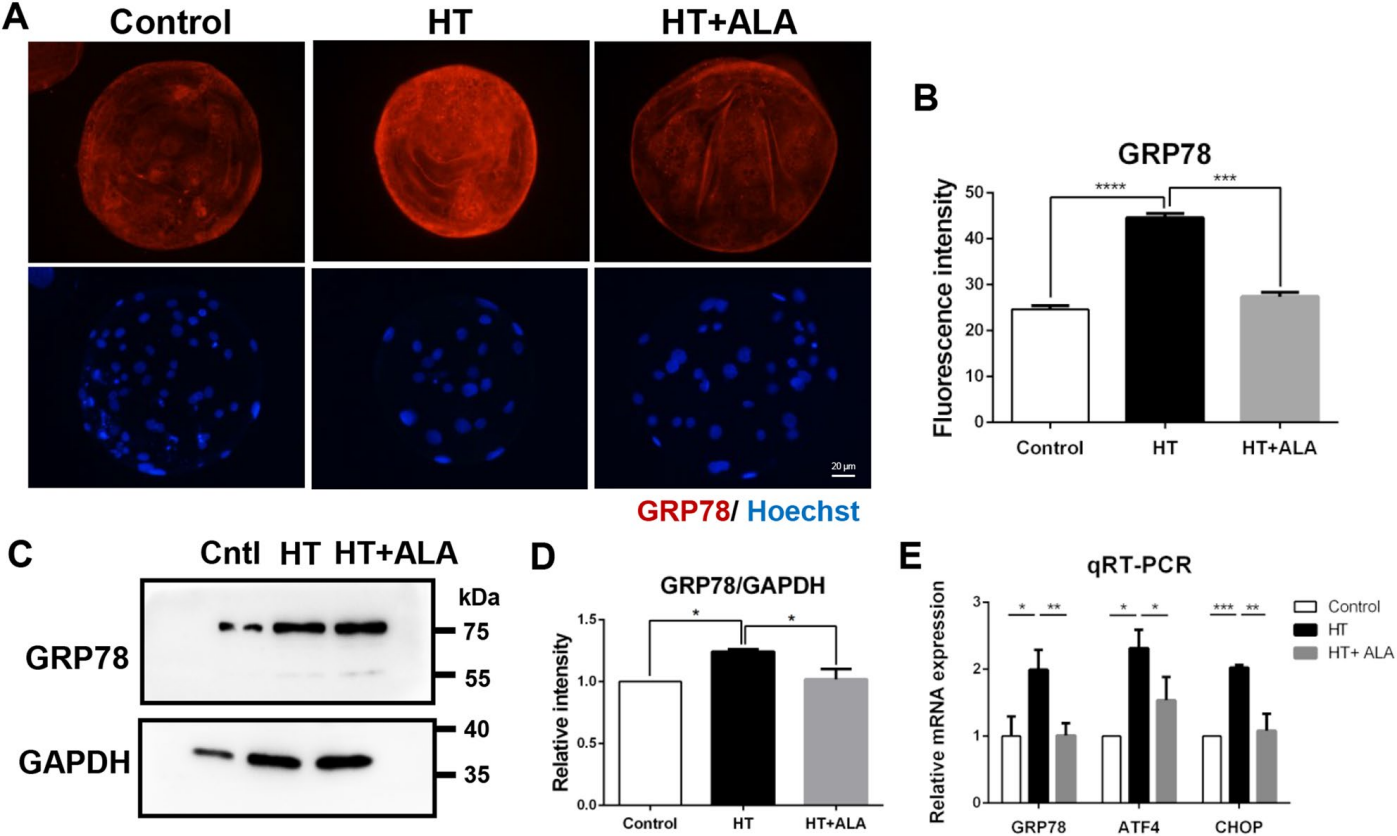
Effect of ALA on ER stress in heat stressed-porcine parthenotes. (**A**) Representative images of GRP78 intensity after HT exposure with ALA addition in porcine parthenotes. Red, GRP78; Blue, DNA. (**B**) Fluorescence intensity of GRP78 after HT exposure in the presence of ALA. (**C**) Western blotting of GRP78 protein expression after HT exposure with ALA treatment. Original blots are presented in Supplementary Fig. 1A,A′. (**D**) Relative band intensity analysis for GRP78/GAPDH after HT exposure with supplementation of ALA. (**E**) Relative mRNA expression of GRP78, ATF4, and CHOP among groups. *p < 0.05; **p < 0.01; ***p < 0.001; ****p < 0.0001.

Given the previous results on oxidative stress, we determined whether ALA had mitigating effects on ER stress after HT exposure. First, we measured the expression levels of glucose regulatory protein 78 (GRP78), an ER stress marker, among the groups. In the HT-treated group, the fluorescence intensity of GRP78 was significantly higher than that in the control group. However, treatment with ALA attenuated its expression, indicating a decrease in ER stress under HT (control: 24.62 ± 0.78 vs. HT: 44.60 ± 0.92, *p* < 0.01; HT: 44.60 ± 0.92 vs. HT + ALA: 27.40 ± 0.86, *p* < 0.0001). No differences were observed between the control and ALA groups (Fig. 4A,B). The relative band intensity of GRP78 was also higher in the HT-treated group than in the control group. ALA addition remarkably reduced its band intensity compared to the HT group (control: 1 vs. HT: 1.24 ± 0.04, *p* < 0.05; HT: 1.24 ± 0.04 vs. HT + ALA: 1.02 ± 0.08, *p* < 0.05, Fig. 4C,D). We also investigated the mRNA expression of other marker genes, such as activating transcription factor 4 (*ATF4*) and C/EBP homologous protein (*CHOP*). The results showed that expression of these marker genes was higher in the HT-treated group, whereas the ALA group exhibited lower expression levels compared to the HT group (GRP78: 1.00 vs. 1.99 vs. 1.01; *p* < 0.05 and *p* < 0.01; ATF4: 1.00 vs. 2.32 vs. 1.54; *p* < 0.05; CHOP: 1.00 vs. 2.02 vs. 1.08; *p* < 0.01and *p* < 0.001, Fig. 4E). Thus, these results suggest that ALA addition attenuates HT-induced ER stress in porcine parthenotes.

### ALA induced HSR in HS-treated parthenotes in pigs

**Figure 5:**
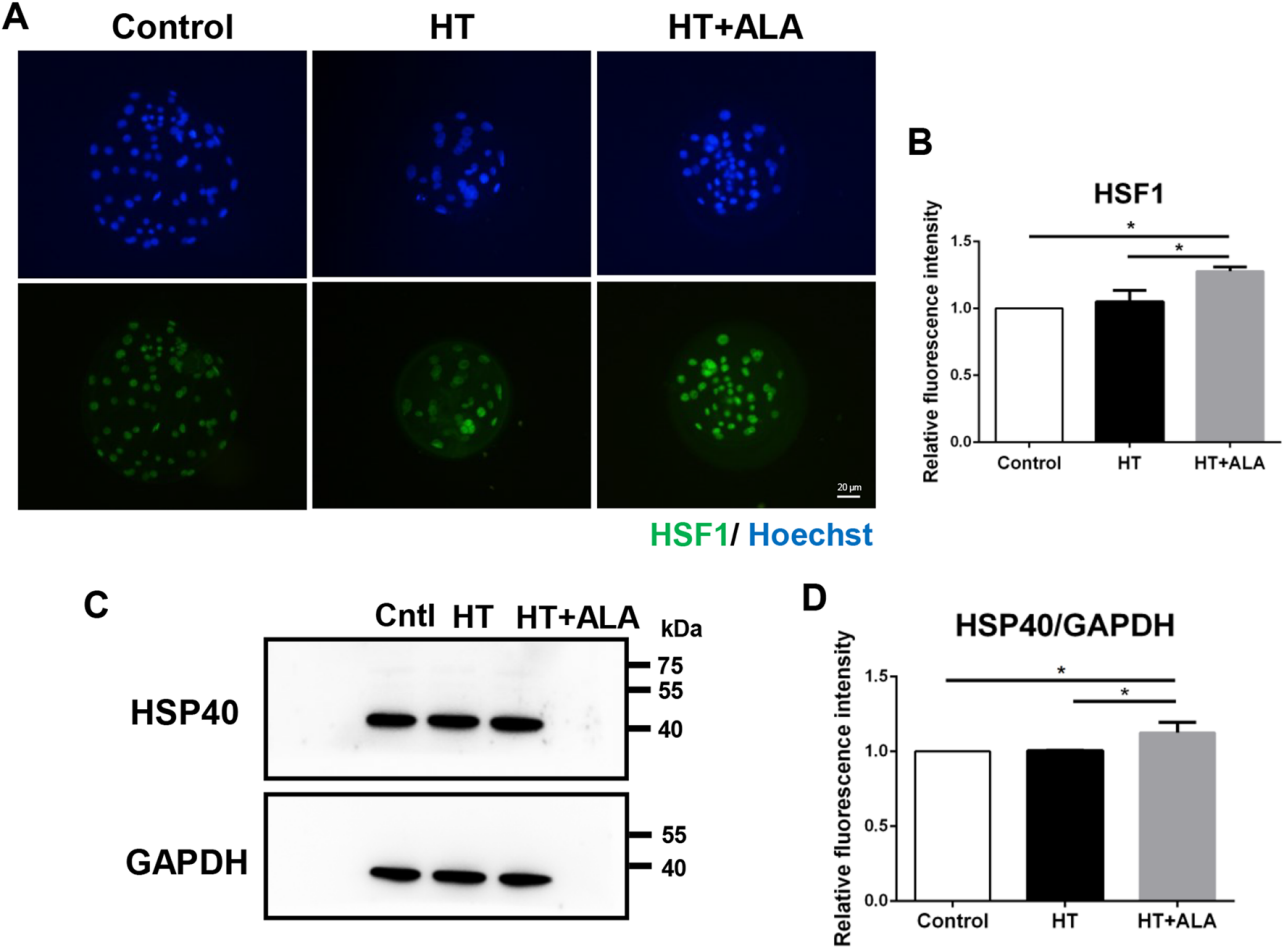
Effect of ALA on the heat shock response in heat stressed-porcine parthenotes. (**A**) Representative images of HSF1 after HT exposure in the presence of ALA in porcine parthenotes. Green, HSF1; Blue, DNA. (**B**) Relative fluorescence intensity of HSF1 after HT exposure with ALA addition. (**C**) Western blotting of HSP40 protein expression after HT exposure with ALA treatment. Original blots are presented in Supplementary Fig. 1B,B′. (**D**) Relative band intensity analysis for HSP40/GAPDH after HT exposure with supplementation of ALA. *p < 0.05.

HT exposure induces ER stress and results in an increase in damaged proteins. Based on the observed effect of ALA treatment on ER stress, we explored the effect of ALA on the HSR under HT conditions. As shown in Fig. 5A,B, the fluorescence intensity of heat shock factor 1 (HSF1) was significantly increased in the ALA treatment group compared to the other groups (control: 1 vs. HT + ALA: 1.26 ± 0.12, *p* < 0.05; HT: 1.05 ± 0.2 vs. HT + ALA: 1.26 ± 0.12, *p* < 0.05). The relative band intensity of HSP40 was also higher in the ALA group than in the other groups (control: 1 vs. HT + ALA: 1.13 ± 0.14, *p* < 0.05; HT: 1.01 ± 0.03 vs. HT + ALA: 1.13 ± 0.14, *p* < 0.05, Fig. 5C,D). Therefore, these results suggest that ALA addition induces an HSR to restore unfolded proteins under HT exposure in porcine parthenotes.

### ALA attenuated apoptosis induced by HS in porcine parthenotes

**Figure 6:**
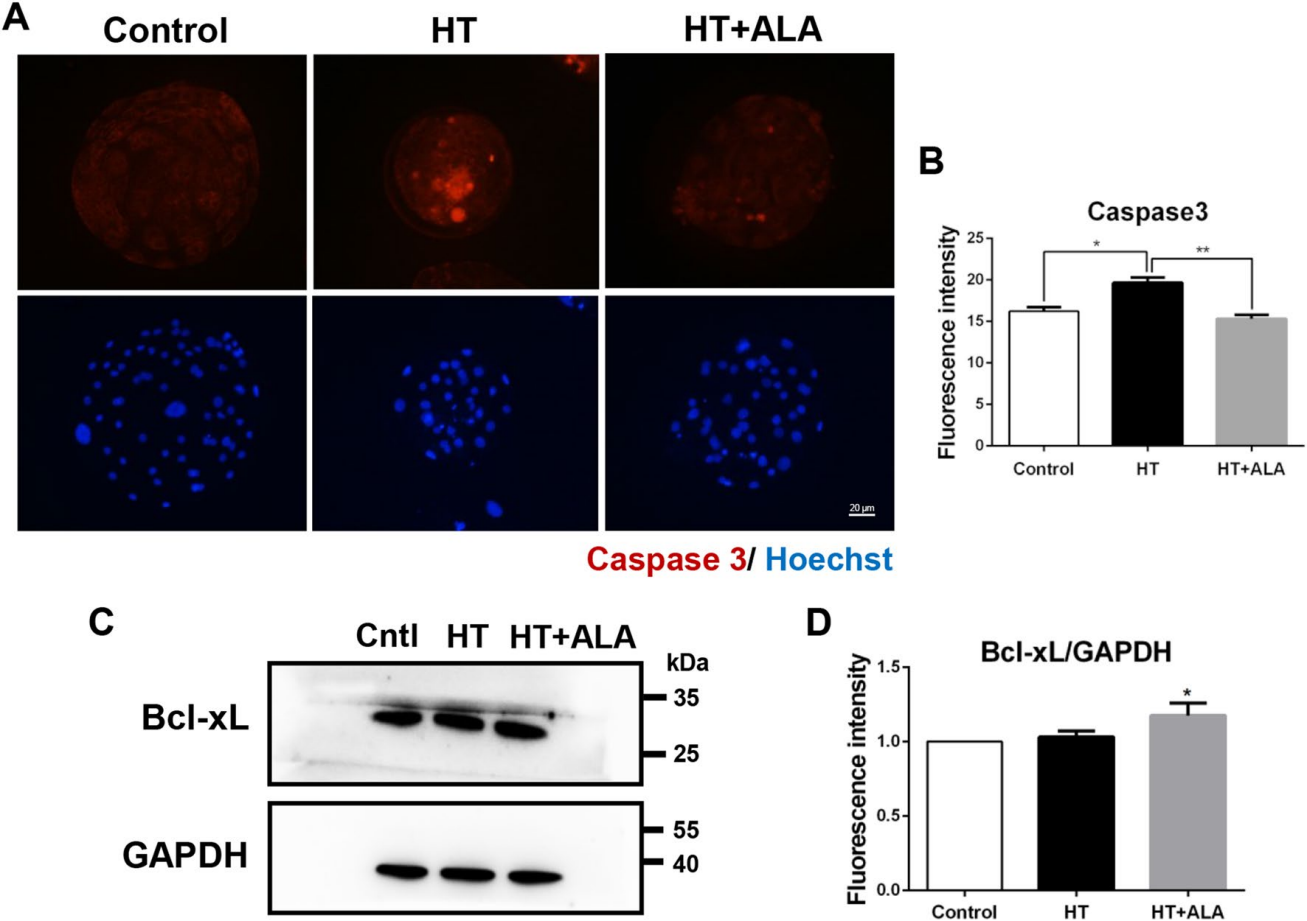
Effect of ALA on apoptosis in heat stressed-porcine parthenotes. (**A**) Representative images of caspase3 after HT exposure in presence of ALA in porcine parthenotes. Red, caspase3; Blue, DNA. (**B**) Relative fluorescence intensity of caspase3 after HT exposure with ALA addition. (**C**) Western blotting of Bcl-xL protein expression after HT exposure with ALA treatment. Original blots are presented in Supplementary Fig. 1B′,C. (**D**) Relative band intensity analysis for Bcl-xL/GAPDH after HT exposure with ALA supplementation. *p < 0.05; **p < 0.01.

Considering the effects of oxidative and ER stresses, we evaluated the effect of ALA on apoptosis after HT exposure. We examined the expression of apoptotic genes such as caspase 3 and B-cell lymphoma-extra-large (Bcl-xL), which are involved in pro-apoptosis and anti-apoptosis, respectively. As shown in Fig. 6A,B, the fluorescence intensity of caspase 3 was significantly increased in the HT-treated group compared to that in the control group, whereas treatment with ALA restored its expression compared to the HT groups (control: 16.21 ± 0.48 vs. HT:19.65 ± 0.59, *p* < 0.05; HT: 19.65 ± 0.59 vs. HT + ALA: 15.30 ± 0.47, *p* < 0.01). In addition, the relative intensity of Bcl-xL was markedly increased in the ALA-treated group compared to that in the control group, suggesting that ALA suppressed apoptosis after HT exposure (control: 1 vs. HT + ALA: 1.18 ± 0.15, *p* < 0.05, Fig. 6C,D). Thus, these results indicate that ALA treatment attenuated apoptosis induced by HT exposure in porcine parthenotes.

## Discussion

**Figure 7:**
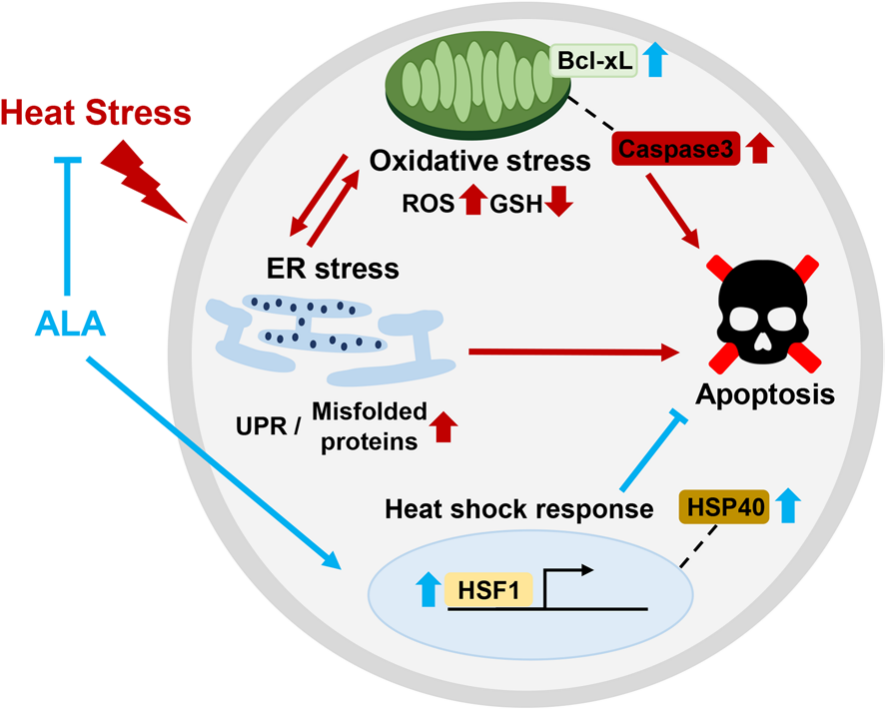
Schematic diagram illustrating the ALA-mediated rescue of HS-induced impairment of parthenotes development. HT exposure induces oxidative and ER stresses, leading to an increase in unfolded proteins, which in turn leads to apoptosis. However, ALA treatment partially ameliorates oxidative and ER stresses and induces the HSR under HS conditions. Overall, ALA treatment attenuates apoptosis after HT exposure.

The therapeutic properties of ALA against cellular stress have been previously described^10,16^. In the present study, we demonstrated that ALA significantly ameliorated the adverse effects of HS by decreasing cellular stress during porcine parthenotes development. Our results show that exposure to HT at 42 °C for 10 h reduced the rate of parthenotes development and its quality by decreasing the total cell number of blastocysts. In addition, HS conditions induced oxidative and ER stresses following an increase in unfolded proteins, which led to apoptosis caused by the increased expression of caspase3. In contrast, treatment with ALA partially ameliorated the oxidative and ER stresses induced by HS and also upregulated the HSR through higher expression levels of HSF1 and HSP40, which increased the refolding of unfolded proteins. Finally, it inhibited apoptosis under HT exposure by increasing Bcl-xL expression (Fig. 7).

In the current study, HT exposure markedly reduced the rate of parthenotes development in pigs, thereby decreasing the cleavage rate throughout the developmental process. These results indicate that HS reduces the developmental potential of porcine parthenotes. In the 4-cell stage of porcine embryos, important conversions from maternal to zygote gradually occur^19^, called major zygotic genome activation. Epigenetic reprogramming can be influenced by increased ambient temperature, and maternal HS leads to global DNA methylation, which reduces antioxidant competence^20^. In addition, early embryos are extremely vulnerable to HS because they develop thermotolerance from the 2-cell stage to the morular stage^21^. HS in the 2-cell stage compromises the development of bovine embryos^22^. To assess the possible protective effect of ALA on heat-stressed porcine parthenotes, we compared three groups: control, HT 10 h, and HT + ALA. The results show that treatment with ALA partially restored the rate of parthenotes development and its quality compared to the HT group, resulting in an increase in the diameter of blastocysts, higher total cell numbers, and fewer TUNEL-positive cells. ALA addition increases the ratio of inner cell mass cells to total cells in blastocysts, suggesting that it may improve the quality of goat embryos^23^. In addition, to confirm whether ALA inhibited apoptosis in blastocysts, we detected the expression of the pro-apoptotic and anti-apoptotic genes caspase 3 and Bcl-xL, respectively. The results show that ALA treatment not only reduced the expression of caspase 3 compared to the HT group but also increased the expression of Bcl-xL compared to the other groups. ALA supplementation improves the quality of blastocysts exposed to ethanol, resulting in fewer TUNEL-positive cells, and decreases expression of apoptotic genes in ovine oocytes^24^. Given the previous results showing a decrease in oxidative and ER stresses, ALA treatment markedly inhibited apoptosis under HS conditions. Additionally, the present results showed a decrease in DNA damage followed by fewer TUNEL-positive cells in the HT group in the presence of ALA. In previous reports, relieving ER stress and promoting HSR have been regarded as potential therapeutic responses against several chronic diseases^25,26^. In addition, HSF1 induces the expression of HSP40 and HSP70 and stabilizes Bcl-xL as a protective response^27^. Therefore, these results reveal the potential benefits of ALA as a therapeutic agent in heat-stressed porcine parthenotes by inhibiting apoptosis.

Oxidative damage induced by HS leads to apoptosis with excess production of ROS^5^. To evaluate whether ALA can attenuate oxidative stress induced by HS in porcine parthenotes, we first detected ROS levels among the three groups. Although HT exposure induced ROS overproduction compared to the control, ALA treatment markedly reduced ROS levels compared to HT treatment. Moreover, higher GSH levels were observed in the ALA group than in the HT group, suggesting that it has strong antioxidant ability. GSH is mainly involved in scavenging ROS during the oocyte stage as a primary antioxidant component^28^. Several antioxidants have been shown to increase GSH levels and diminish ROS levels in oocytes exposed to HS. This suggests that the modulation of antioxidants under HS conditions may relieve thermal-oxidative stress in oocytes and enhance fertility^5^. In addition, ALA dissolves in both water and lipids, which enables it to freely pass through biological membranes, and generally serves as an antioxidant in the cytosol, extracellular spaces, and plasma, thereby effectively protecting cells against ROS injury^29^. Thus, these results demonstrate that ALA ameliorates ROS production by regenerating GSH, resulting in a decrease in oxidative stress under HS conditions.

A few pathological signal transduction pathways, as well as ER stress and apoptosis, are promoted by oxidative stress^30^. Accumulation of unfolded or aggregated proteins activates ER stress, which induces many chaperones to restore these proteins, regarded as the UPR pathway^31^. Although the UPR protects cells from several stresses and exerts its effects on protein homeostasis, prolonged ER stress can induce cell death^30^. Based on our results regarding oxidative stress after HT exposure, we further examined ER stress and evaluated the curative effect of ALA under HT exposure. Our results revealed that HT exposure resulted in higher protein expression of GRP78 and mRNA levels of GRP78, ATF4, and CHOP, which are ER stress markers, indicating the occurrence of ER stress. ALA supplementation markedly reduced the expression of all marker genes and GRP78 protein levels in porcine parthenotes, suggesting that it inhibited ER stress under HS conditions. In previous studies, thermal stress stimulated the formation of protein aggregates in the ER, resulting in oxidative stress-related damage^31^. ALA has the potential to regenerate endogenous antioxidants such as vitamin C, vitamin E, and GSH and is attracting attention as a therapeutic support to ameliorate ER stress^32^. ALA protects against cadmium-induced ER stress in rats and attenuates heat-damaged injury by inhibiting ER stress in chicken testes^10,33^. Taken together, these results suggest that ALA attenuates ER stress in HS-induced porcine parthenotes.

Interestingly, we found that HT exposure in the presence of ALA induced the HSR in porcine parthenotes, indicating an increase in the expression of HSF1 and HSP40. The HSR is induced by HS or oxidative damage, and HSPs serve as molecular chaperones and protect against misfolded or aggregated proteins^34^. Therefore, heat damage immediately activates HSFs, which leads to the synthesis of HSP70 and HSP40 as cell survival signaling molecules^35^. However, in the present study, there were no differences in the expression of HSF1 and HSP40 between the control and HT groups. ER stress induced by thermal stress suppresses HSR via translational blockade in rats^35^. ALA has a beneficial effect on modulating diverse components of the HSR including HSP25, HSP72, and HSF1, which results in the attenuation of ER stress and improvement in insulin sensitivity^36^. In addition, ALA prevents heat stroke-induced myocardial damage by acting as an antioxidative and anti-inflammatory agent with the induction of HSP70^37^. Thus, based on the previous results on ER stress recovery by ALA treatment, these results suggest that ALA supplementation induces HSR in parallel with suppression of ER stress in heat-stressed porcine parthenotes.

In conclusion, ALA ameliorated HT-induced apoptosis by suppressing oxidative and ER stresses by inducing the HSR in porcine parthenotes. Moreover, these results revealed that ALA had a strong protective function against HS in pigs and demonstrate its therapeutic role in protein stabilization via activation of the HSR.

## Materials and methods

All chemicals were purchased from Sigma-Aldrich (St. Louis, MO, USA) unless otherwise indicated, and all animal studies were conducted following the guidelines of the Institutional Animal Care and Use Committee (IACUC) of Chungbuk National University, South Korea. In present study, parthenogenetic diploids were used due to the relatively high occurrence of polyspermy with in vitro fertilization of porcine embryos. However, the development of porcine parthenogenetic diploids to the blastocyst stage is comparable to the normal development of embryos^38–40^.

### Oocyte collection and in vitro maturation

Porcine ovaries were acquired from a local slaughterhouse (Farm Story Dodram B&F, Umsung, Chungbuk, South Korea) and transported to the laboratory at 38.5 °C in saline. Cumulus–oocyte complexes (COCs) were aspirated from ovarian follicles (3–6 mm in diameter), and oocytes enclosed by at least three layers of cumulus cells were collected for further experiments. After washing thrice with Tyrode lactate HEPES (TL-HEPES) buffer, and the COCs were transferred to an in vitro maturation medium containing TCM-199 (Invitrogen, Carlsbad, CA, USA), supplemented with 10% (v/v) porcine follicular fluid, 0.91 mM sodium pyruvate, 0.6 mM l-cysteine, 10 ng/mL epidermal growth factor, 10 μg/mL luteinizing hormone, and 0.5 μg/mL follicle-stimulating hormone, and were cultured for 44 h at 38.5 °C in a humidified 5% CO_2_ incubator.

### Parthenogenetic activation and in vitro culture

Parthenogenetic activation and in vitro culture were conducted as previously reported^41^. After removing the cumulus cells by repeated pipetting in 1 mg/mL hyaluronidase, denuded oocytes were parthenogenetically activated by two direct-current pulses of 120 V for 60 µs in 297 mM mannitol (pH 7.2) containing 0.1 mM CaCl_2_, 0.05 mM MgSO_4_, 0.01% polyvinyl alcohol (PVA, w/v), and 0.5 mM HEPES. Then, these oocytes were incubated in bicarbonate-buffered porcine zygote medium 5 (PZM‐5) containing 4 mg/mL bovine serum albumin (BSA) and 7.5 µg/mL cytochalasin B for 3 h to inhibit extrusion of the pseudo-second polar body. Next, the oocytes were thoroughly washed and incubated in bicarbonate‐buffered PZM‐5 supplemented with 4 mg/mL BSA in 4-well plates for 7 days at 38.5 °C in humidified atmosphere containing 5% CO_2_. Four-cell cleavage rate and morula and blastocyst formation rates were recorded at 48, 96, and 144 h after activation, respectively. Blastocyst diameter was examined in parthenotes at the blastocyst stage on day 7 using ImageJ v.l.44 g software (National Institutes of Health, Bethesda, MD, USA).

### TUNEL assay

The intracellular apoptosis standard of blastocysts was determined with TUNEL assay using an In Situ Cell Death Detection Kit (11684795910; Roche, Basel, Switzerland), as described earlier^42^. Blastocysts were fixed in 3.7% formaldehyde for 30 min at room temperature (RT) and permeabilized by incubation in 0.5% Triton X-100 for 30 min at RT. Next, the blastocysts were cultured with fluorescein-conjugated dUTP and terminal deoxynucleotidyl transferase enzyme for 1 h at 38.5 °C. After washing thrice with phosphate-buffered saline/polyvinyl alcohol (PBS/PVA), the blastocysts were treated with 10 µg/mL Hoechst 33,342 for 10 min and mounted onto glass slides. Images were obtained using a confocal microscope (LSM 710 Meta; Zeiss, Oberkochen, Germany). The apoptosis index was calculated by dividing the TUNEL-positive cell number by the total cell number.

### GSH and ROS measurements

To determine GSH levels, blastocysts were cultured for 30 min at 38.5 °C in PBS/PVA containing 10 μM 4-chloromethyl-6,8-difluoro-7-hydroxycoumarin dye (CellTracker™ Blue CMF_2_HC, Thermo Fisher Scientific, Waltham, USA) as previously described^43^. To determine total ROS levels, blastocysts were incubated for 30 min at 38.5 °C in PBS/PVA containing 10 μM 2′,7′-dichlorodihydrofluorescein diacetate (H_2_DCF-DA, Cat # D399, Molecular Probes, Eugene, OR, USA). After incubation, the blastocysts were washed thrice with PBS/PVA. Fluorescence signals were detected as TIFF files using a digital camera (DP72; Olympus, Tokyo, Japan) connected to a fluorescence microscope (IX70; Olympus). GSH and ROS levels were quantified by analyzing the fluorescence intensity in blastocysts using ImageJ v.1.44g software (National Institutes of Health, Bethesda, MD, USA).

### Immunofluorescence staining

Immunostaining was conducted as previously described^41^. After washing thrice with PBS/PVA, blastocysts were fixed with 3.7% formaldehyde at RT for 30 min. Next, blastocysts were permeabilized in 0.5% Triton X-100 for 30 min at RT, and incubated in PBS/PVA containing 3.0% BSA at RT for 1 h. Subsequently, these blastocysts were incubated overnight at 4 °C with rabbit anti-GRP78 (1:100; ab21685, Abcam, Cambridge, United Kingdom), rabbit anti-HSF1 antibody (1:100; 12972S, Cell Signaling, Danvers, MA, United States), and rabbit anti-caspase3 antibodies (1:20; 9664S, Cell Signaling). After washing thrice with PBS/PVA, the blastocysts were incubated with Alexa Fluor 488™ goat anti-rabbit IgG (1:200; A32731, Invitrogen) or Alexa Flour 546™ donkey anti-rabbit IgG (1:200; A10040, Invitrogen, Carlsbad, CA, United States) at 37 °C for 1 h. After three washes, the blastocysts were incubated for 10 min with 5 μg/mL Hoechst 33342. Finally, the blastocysts were mounted on slides and examined under a confocal microscope (LSM 710 META; Zeiss, Oberkochen, Germany). Images were processed using Zen software (v.8.0; Zeiss, Jena, Germany) and then analyzed using ImageJ v.l.44 g software (National Institutes of Health, Bethesda, MD, USA).

### Quantitative reverse transcription-polymerase chain reaction (qRT-PCR)

As previously described^42^, mRNA was extracted from 30 embryos in each group using a Dynabeads mRNA Direct Kit (61012; Thermo Fisher Scientific, Waltham, MA, USA), and cDNA was obtained using a First Strand Synthesis Kit (cat# 6210; LeGene, San Diego, CA, USA) following the manufacturer’s instructions. qRT-PCR was performed using a WizPure qPCR Master mix (W1731-8; Wizbio Solutions, Seongnam, South Korea) according to the manufacturer’s instructions, using a QuantStudio™ 6 Flex Real-Time PCR System (Applied Biosystems, Waltham, MA, USA). Amplification was performed as follows: initial denaturation at 95 °C for 10 min, followed by 40 cycles of amplification at 95 °C for 15 s, 60 °C for 20 s, and 72 °C for 15 s, and a final extension at 95 °C for 15 s. Relative gene expression was calculated using the ∆∆CT method. The primers used to amplify each gene are listed in Table 1.

**Table 1:** Primer sequences used in RT-qPCR.

Gene	Gene accession no	Primer sequence (5′–3′)	Annealing temp (°C)	Length (bp)
*GRP78*	J03214.1	F: ACC AAT GAC CAA AAT CGC CT R: GTG ACT TTC CAG CCA CTC AA	60	246
*ATF4*	NM_001123078.1	F: TGA GCC CTG ACT CCT ATC TG R: TCC AGC TCT TTA CAT TCG CC	60	277
*CHOP*	NM_001144845.1	F: AAG ACC CAG GAA ACG GAA AC R: TCC AGG AAA GGT CAG CAG TA	60	261
*GAPDH*	AK234838	F: AAGTTCCACGGCACAGTCAAG R: CACCAGCATCACCCCATTT	60	112

### Western blotting analysis

As previously reported^43^, 80 porcine embryos from each group were collected in sodium dodecyl sulfate sample buffer and heated at 95 °C for 5 min. Proteins were separated by sodium dodecyl sulfate–polyacrylamide gel electrophoresis and electrotransferred to polyvinylidene fluoride membranes. Next, the membranes were blocked in Tris-buffered saline containing Tween 20 (TBST) with 5% skim milk for 1 h and incubated overnight at 4 °C with rabbit anti-GRP78 antibody (1:1000; ab21685, Abcam), rabbit anti-HSP40 antibody (1:1000; ab69402, Abcam), rabbit anti-Bcl-xL antibody (1:1000; #2762, Cell Signaling), and rabbit anti-GAPDH antibody (1:1000; 5174S, Cell Signaling). After washing thrice with TBST, the membranes were incubated with horseradish peroxidase-conjugated goat anti-mouse IgG or goat anti-rabbit IgG (1:2000) at RT for 1 h. The blots were cut prior to hybridization with antibodies and have the absence of images of full length (Supplementary Fig. 1). Signals from blots were captured using a charge-coupled device camera and UviSoft software (Uvitec, Cambridge, United Kingdom).

### Experimental design

To ensure parthenotes of steady quality, the 2-cell cleavage rate was confirmed after 24 h of parthenogenetic activation. Parthenotes of the one-cell and two-cell stages and the same quality were separated evenly into three groups: control, HT, and HT + ALA (10 µM). ALA was diluted in ethanol 0.1% (the toxicity of which has been previously evaluated^44^ and added at 10, 15, or 20 µM to porcine parthenotes cultured in vitro. The concentration of ALA was selected based on the results. The control group was cultured at 38.5 °C. HT and HT + ALA groups were cultured at 42 °C for 10 h and then returned to 38.5 °C^45^ and continuously cultured for 6 days.

### Statistical analysis

Each experiment was performed three times, each sample in triplicates. Data were analyzed using one-way analysis of variance (ANOVA) or the Student’s *t*-test. All percentage data were subjected to arcsine transformation before statistical analysis and are presented as mean ± SEM. Differences were considered statistically significant at *p* < 0.05. All calculations were performed using the GraphPad Prism 6 software (GraphPad, San Diego, CA, USA).

## Supplementary Information

Supplementary Information.

## Data Availability

The datasets used or analyzed during the current study are available from the corresponding author upon reasonable request.
