# Supplementary figures and images for: Alpha-lipoic acid attenuates heat stress-induced apoptosis via upregulating the heat shock response in porcine parthenotes
Source: Sci Rep. 2023 May 24;13:8427. doi: 10.1038/s41598-023-35587-6 (PMC10209172; doi:10.1038/s41598-023-35587-6)

Supplementary Figure 1. Uncropped Western blots data.

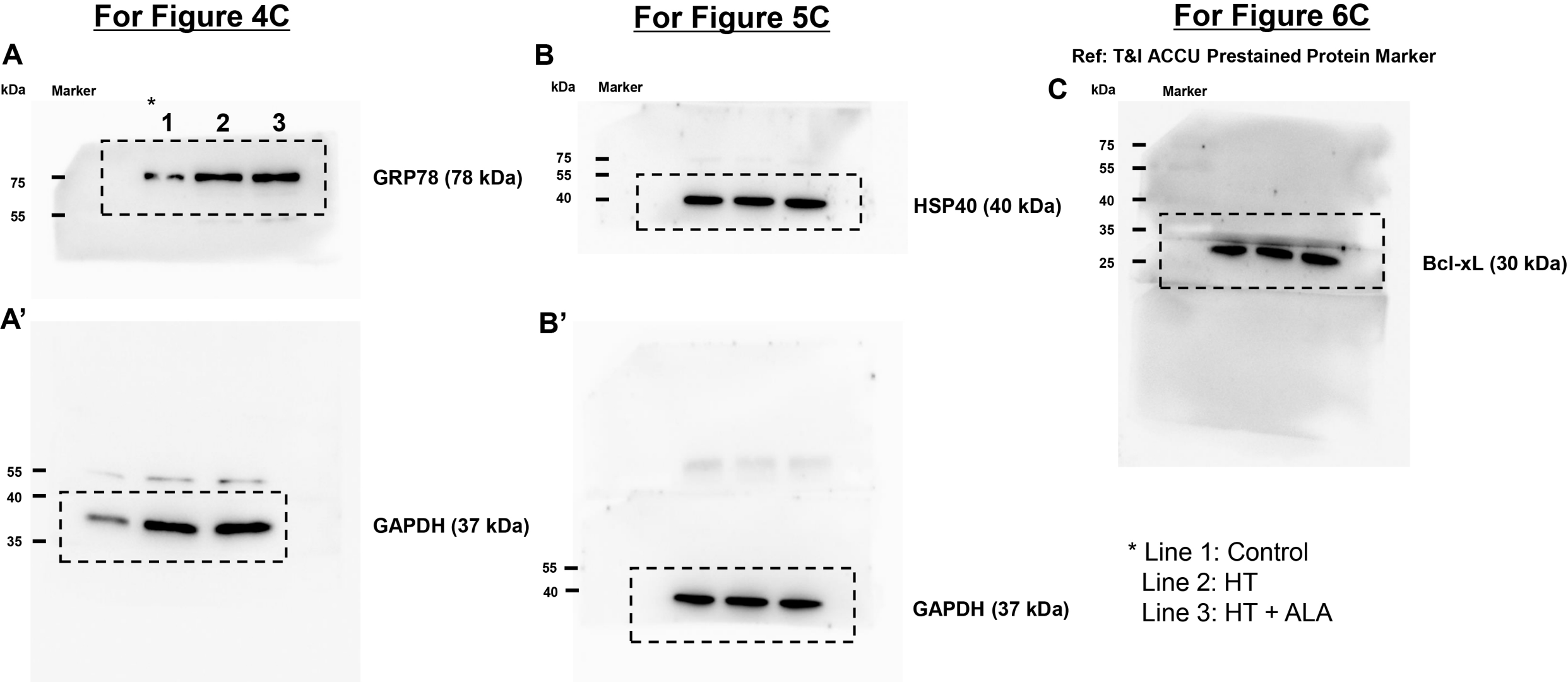

Supplement: Supplementary file 1 — Supplementary Information. [file 41598_2023_35587_MOESM1_ESM.pdf]
